# Who uses what food retailers? A cluster analysis of food retail usage in the Netherlands

**DOI:** 10.1016/j.healthplace.2023.103009

**Published:** 2023-04-10

**Authors:** Jody C. Hoenink, Milou Eisink, Jean Adams, Maria G.M. Pinho, Joreintje D. Mackenbach

**Affiliations:** 1 MRC Epidemiology Unit, University of Cambridge School of Clinical Medicine, Box 285 Institute of Metabolic Science, Cambridge Biomedical Campus, Cambridge CB2 0QQ, UK; 2 Upstream Team, https://www.upstreamteam.nl/, Amsterdam UMC, The Netherlands; 3 Amsterdam UMC, Vrije Universiteit Amsterdam, Epidemiology and Data Science, Amsterdam Public Health Research Institute, De Boelelaan 1117, Amsterdam, the Netherlands; 4 Copernicus Institute of Sustainable Development, Department Environmental Sciences, Utrecht University, Utrecht, the Netherlands

**Keywords:** Food supply, dietary behaviors, food environment, exposure to food outlets, residential environment

## Abstract

The aim of this study is to describe how individuals use different food retailers and how food retail usage varies according to socio-demographic and diet-related characteristics. A cross-sectional survey among Dutch adults (N = 1784) was used. Results from the Two-step cluster analysis indicated that there were five clusters of food retail users. Use of discount supermarkets, organic supermarkets, fast-food outlets, and restaurants contributed to clustering, but use of regular supermarkets, local food shops and whether food retailers were close to home or further from home did not. The clusters included mixed food outlet users, discount supermarket and restaurant users, fast-food and restaurant users, predominant discount supermarket users and supermarkets, fast-food and restaurant users. Participants in each cluster had their own characteristics especially in terms of socio-economic position and diet quality. Future studies need to consider further how food retail selection links physical exposure to the food environment and diet.

## Introduction

Worldwide, rapid urbanisation and globalisation have led to environments where foods high in fat, salt and sugar are now increasingly easily accessible [1, 2]. Indeed, several studies including our own have demonstrated significant changes in urban food environments in the last decade [3–5]. These increases in access are paralleled by increases in consumption of fat, salt and sugar [2].

Exposure to the food environment is usually defined in geographical terms, by measuring access to or availability of food retailers from a person's home address [6, 7]. However, reported associations between local food environments, dietary intake and obesity are inconsistent [6, 7]. Some studies show that exposure to less healthy food retailers (e.g. fast food restaurants and convenience stores) is associated with decreased dietary quality and increased risk of obesity [8, 9], while most studies show null effects [6, 10–12], and some even find that exposure to less healthy food retailers is associated with healthier diets [6, 13].

Greater clarity on the influence of food environments on diet and health outcomes can help researchers identify strategies to improve health. Though it is possible that the local food environment does not influence diet and obesity, a limitation of the current literature base is that the assumptions underlying the exposure-outcome association remain largely untested. In operationalizing ‘exposure’ to the food environment, assumptions are made around the size and shape of the geographic unit of interest (e.g. in which directions and how far people travel to food retailers [14]) and the interaction between individuals and their environment (e.g. how many and what food retailers are used) [15]. Individuals do not necessarily shop at the food retailers most proximal to their home, suggesting that there are reasons other than distance for choosing a food retailer [11, 16]. Also, food retail exposure not only takes place around the home environment, but also in work, school and recreational settings [17]. Caspi et al. [7] conclude that 'a tremendously understudied aspect of food retail access is the utilization of food retailers by area residents'. Additionally, in an attempt to simplify a complex reality, researchers often resort to a dichotomization (e.g. healthy vs. less healthy) or selection (e.g. only fast-food outlets) of food retailers when considering the food environment [6, 18]. Because food retail exposure and use do not operate in a vacuum, simultaneous consideration of exposure to food retailers and their use is important [19]. Common metrics to capture the complexity of the food environment include the use of ratios and relative measures. However, the identification of clusters of food retail users may be preferable [20].

Some previous studies assessed the assumptions underlying the exposure-outcome association by investigating the spatial locations of food purchasing behaviours [16, 17]. However, few previous studies have explored the clustering of multiple food retailers in the general population [21–23]. An older study by Carlson et al. [21] grouped consumers based on where they obtained their food and found nine clusters. The largest cluster including 49% of participants, consisted of those that purchased 93% of their food from supermarkets. Individuals in this cluster were older and had a lower socio-economic position (SEP) compared to individuals in the other clusters. Similarly, Yenerall et al. [22] found that all six clusters of food retail use were dominated by retailers selling foods to cook at home (e.g. grocery stores and supermarkets). This study also found significant differences in terms of sociodemographic characteristics and taste preferences between clusters. The study by Stern et al. [23] found three clusters (primary-grocery, primary-mass-merchandise and combination clusters). However, this study only included retailers conducive to food at home limiting generalisability to the wider food environment and total food intake [22]. Furthermore, all of these studies were conducted in the United States (US) and none of these studies explored where food retail use took place or assessed the diet quality of the individuals in the different clusters. Usage of different types of food retailers could influence dietary quality, or individuals with healthier diets could choose to shop more frequently at certain types of food retailers [24]. Furthermore, as all studies were conducted in the US, these findings may not be generalizable to other countries.

The aim of this study was to enhance understanding of how individuals interact with the food environment by investigating the extent to which there are distinct clusters of food retailer use in the Netherlands. In addition, we aimed to investigate the socio-demographic and diet-related characteristics of individuals in each food retail cluster. As the total impact of the food environment includes food availability in various settings such as neighbourhoods surrounding home, work and travel paths, we distinguished between food retail use in the residential neighbourhood environment and further away.

## Methods

This cross-sectional study used data from the ‘Eet & Leef’ study, which aimed to assess dietary intake and its determinants among the Dutch general population (aged 18-65 years). Full details of the study are described elsewhere [25, 26]. Briefly, participants were recruited from the 20 largest urban cities in the Netherlands and were recruited online and via mail through a stepwise recruitment approach in 2019 [26]. Participants were included in the study if they were between the ages of 18 and 65 years and provided informed consent. Participants were excluded if they were not able to understand the Dutch language or did not have access to a computer with internet and an e-mail address. Instead of one long survey, participants were asked to complete three separate web-based surveys over a period of 2-3 weeks. Participants who completed all three surveys received a gift voucher of €7.50. In this analysis, we used data from the first survey containing questions on socio-demographics, psychosocial resources, lifestyle and health, snacking behaviours and perceptions of the food environment and from the third survey on diet quality. No variables from the second survey were used in this study. In total, 2552 participants registered for the study, 1784 participants completed the first survey, 1659 completed the second survey and 1492 participants completed all three surveys. The study was approved by the Medical Ethics Review Committee.

### Measures

#### Food retail use

Participants provided information on their use of six types of common food retailers in the Netherlands: regular supermarkets, organic supermarkets, discount supermarkets, specialist shops (e.g. bakery, greengrocer, butcher), fast-food outlets (e.g. local fast-food shops, McDonalds, Kentucky Fried Chicken, Burger King), and other restaurants. Table 1 displays the questions and answer possibilities participants received that established food retail use and how these were coded in analysis. In total, twelve dichotomous variables were included relating to the use of the six food retailers within a 10-minute walk or further away from home.

#### Socio-demographic characteristics

Several socio-demographic characteristics that have been linked to dietary behaviour (e.g. [27, 28]) were assessed: age in years, sex (male/female), partner status (yes/no), number of children living in the household (regardless of age), educational level, occupation and income. Information on the SEP proxies educational level, occupation, and income have been previously published [25]. Briefly, low educational level included those who completed no education, primary education, lower vocational education and general secondary education. Medium education included those who completed secondary vocational education or intermediate vocational education and high educational level included those who completed higher professional education (College/University). Occupation was classified using the ISCO08 [29]. Skill level 1 included occupations labelled as simple and routine physical or manual tasks. Skill level 2 occupations include tasks such as operating machinery. Skill level 3 are those that involve the performance of complex technical skills, and skill level 4 occupations require complex problem-solving, decision-making and creativity. Net monthly household income was assessed using the question ‘What is your net household income (after tax deduction) per month’ and included five answering options (ranging from €0-€1200 to more than €4000).

#### Diet-related behaviours

Behaviours that have been previously linked to diet quality were also assessed [30–35]. Participants were asked how frequently they consumed 10 different snack foods (Supplementary Table 1; e.g. candy, cookies and ice cream), cooked at home (Supplementary Table 2), ordered dinner online (Supplementary Table 2) and ordered groceries online (answer options ranging from never to 2x per week or more). Grocery shopping style was assessed by asking participants about whether they made a shopping list before doing the groceries, if they shopped for groceries once a week and if they decided beforehand what they are going to purchase within the store (Supplementary Table 3; on a 5-point Likert scale ranging from never to always). When describing the diet-related behaviours, answering categories with fewer participants were combined. Thus, the diet-related behaviours included were snacking frequency, cooking frequency, ordering dinners online, ordering groceries online and grocery shopping style.

#### Diet quality

As previously described [25], diet quality was assessed using the Dutch Healthy Diet 2015 index (DHD15-index). The DHD15-index and energy intake was measured using the 34-item Dutch Healthy Diet Food Frequency Questionnaire [36]. The DHD15-index consist of fifteen components, namely vegetables, fruits, whole grain products, legumes, nuts, dairy, fish, tea, fats and oils, coffee, red meat, processed meat, sweetened beverages and fruit juices, alcohol and salt. Each component receives a score ranging from 0 to 10, on which a total score is calculated ranging from 0 (no adherence) to 150 (complete adherence). If participants had implausible energy intake levels (<500kcal or >3500kcal/day for women and <800kcal or >4000kcal/day for men(34)), their data were excluded from the analyses on diet quality (n=32, 1.7%).

#### Statistical analyses

Descriptive statistics were performed for socio-demographic characteristics, diet-related behaviours and diet quality using frequencies and percentage, means with standard deviations or median with interquartile range as appropriate. A Two-Step cluster analysis was used to identify clustering of food retail use. The Two-Step cluster analysis is a hybrid approach which first uses a distance measure to separate groups and then a probabilistic approach (similar to latent class analysis) to choose the optimal subgroup model [37]. We used the Log-likelihood distance measure to group individuals in clusters based on their food retail usage (12 dichotomous variables) as this measure can be used on categorical variables. Descriptive statistics were performed to indicate the presence and usage of food retailers within the cluster solutions.

The number of clusters was determined automatically to reveal natural clusters, using the best combination of low Bayesian Information Criterion (BIC), high ratio of BIC changes and high ratio of distance measure. As it is possible that clustering problems occur in which the BIC continues to decrease as the number of clusters increases [38], the number of clusters was also checked manually by evaluating the changes in BIC and distance measure. This was done by using a relatively large BIC change and large Ratio of Distance Measures [38]. The quality of the cluster solutions were manually determined using the silhouette measures of cohesion and separation, which measure the distance between clusters (score <0.25: no clustering, 0.26–0.50: weak clustering, 0.51–0.70: reasonable clustering, 0.71–1.00: strong clustering) [39]. We used a distance measure between clusters of 0.51 as a cut-off to determine whether there was clustering or not. Additionally, predictor importance was evaluated (ranging from 0 to 1 where lower numbers indicate that the contribution of a food retail variable is less important to the cluster). A pre-determined stepwise approach was used to identify the best cluster solution. A-priori we specified that in the case of weak cluster quality (i.e. a distance measure lower than 0.51), food retail variables within a 10-minute walk and those further away from home would be combined, thereby counting each type of food retailer only once instead of twice. Thus, if participants indicated use of a food retailer within a 10-minute walk or further away from home, this participant was considered to use the food retailer. If low cluster quality was still present after this, we pre-specified that we would drop types of food retailers that had low predictor importance.

As cluster solutions can depend on data ordering, randomly ordering of the dataset is advised to verify the stability of the given cluster solution [40]. As such, a sensitivity analysis was performed by creating three random variables that were used to sort the dataset in random order three different times. All cluster solutions were checked three times with the dataset in these three different random orders. In all three cases, this led to the same cluster solution as the main analyses.

**Figure 1:**
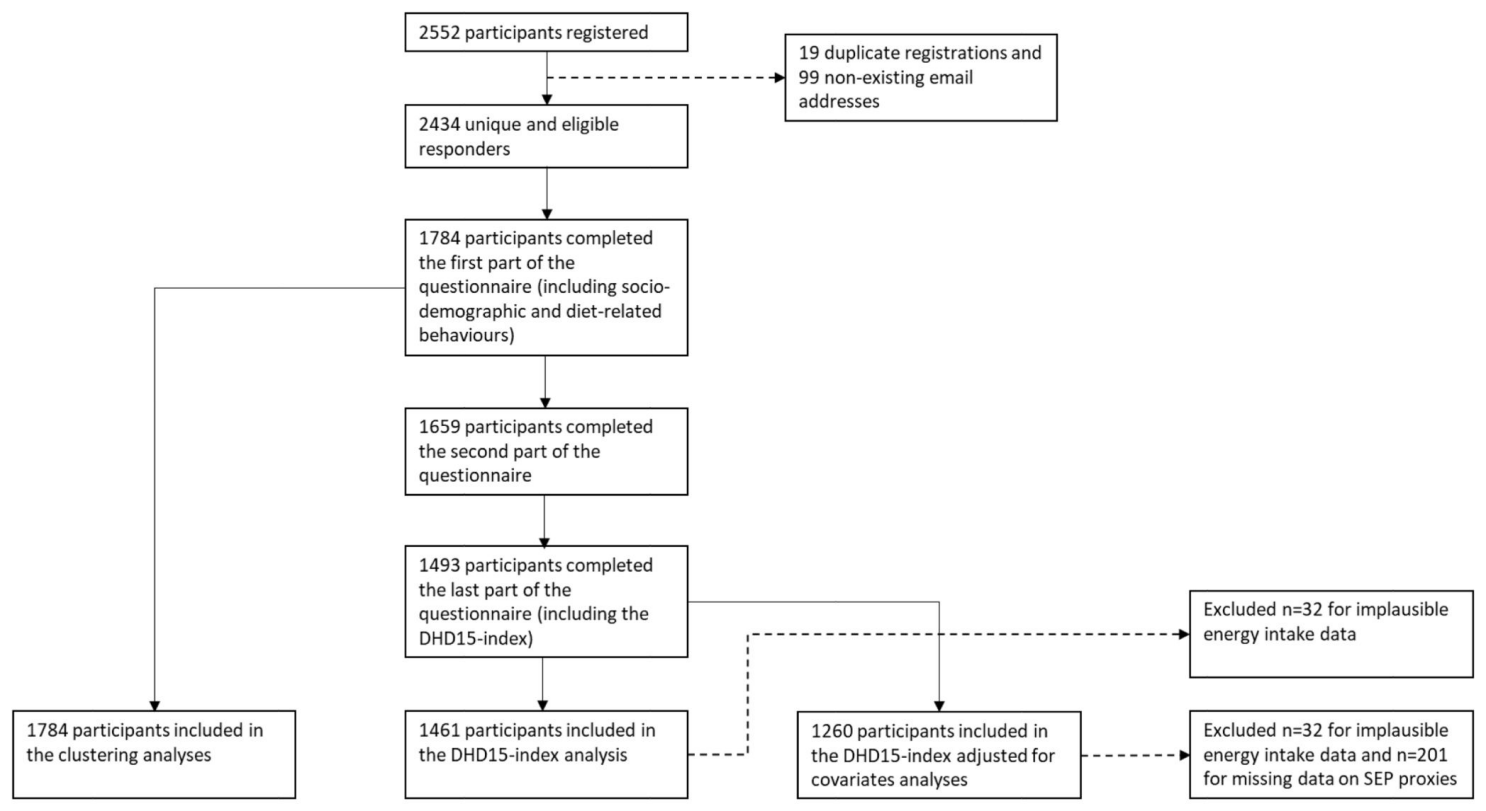
Flow chart of participant inclusion in the Eet & Leef study and the current analyses

Differences between clusters with regards to socio-demographic characteristics, diet-related behaviours and diet quality were analysed using Chi-Squared tests (in the case of categorical variables), analyses of variance (ANOVA in case of normally distributed continuous variables)) or the Kruskal-wallis H test (in case of non-normally distributed continuous variables). In case of statistical significance (i.e. a p-value <0.05), post-hoc analyses were conducted (including the Tukey method for the ANOVA). The General Linear Model Univariate procedure was used to estimate and compare the mean DHD15-index of individuals in the food retail clusters adjusted for covariates (i.e. sociodemographic characteristics and diet-related behaviours). Complete case analyses were used (flow chart displayed in Figure 1) and all analyses, including the Two-Step cluster analysis, were implemented in IBM SPSS Statistics (version 27.0) [41].

## Results

In total, n=1784 participants completed the first survey including information on participants’ socio-demographics, psychosocial resources, lifestyle and health, snacking behaviours and perceptions of the food environment. Missing data was only found for the three SEP indicators. The analytical sample including dietary quality consisted of n=1461 participants. Participant sociodemographic and diet-related characteristics are described in Table 2.

**Figure 2:**
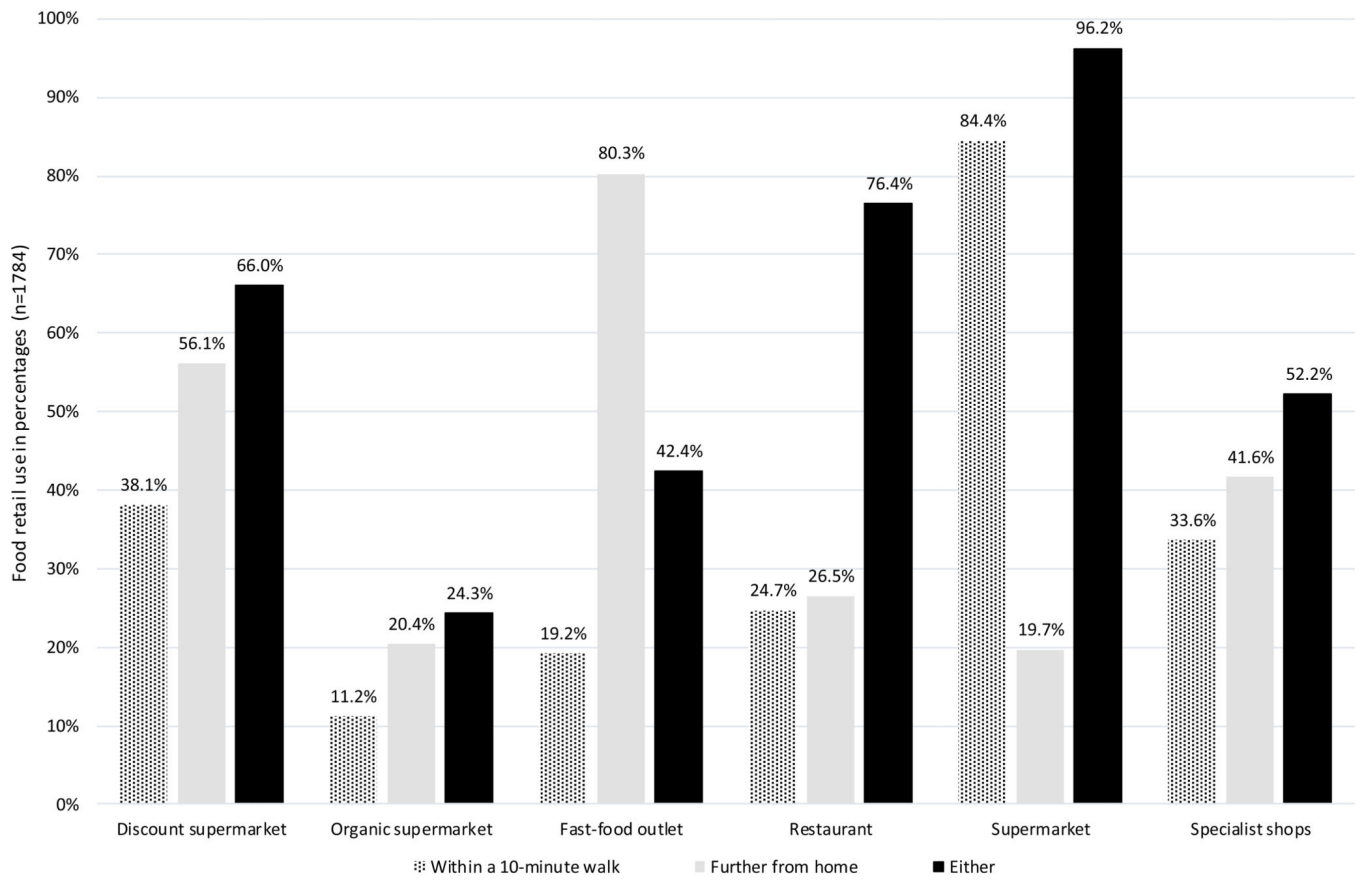
Percentage of study population (N = 1784) who used food retailers within a 10-minute walk, further away from home, and who used either options. As participants are able to shop at multiple food outlets in multiple areas, these percentages do not add up to 100%.

Figure 2 illustrates that the most commonly used food retailer was the supermarket (used by 1716 or 96% of participants regardless of distance), followed by restaurants further from home and discount supermarkets further from home. In general, use of retailers further from home was more common than retailers within a 10 minute walk (e.g. 20% of participants indicated they used organic supermarkets further from home whilst only 11% indicated that they used organic supermarkets within a 10-minute walk). The only exception was for regular supermarkets.

### Cluster profiles

**Figure 3:**
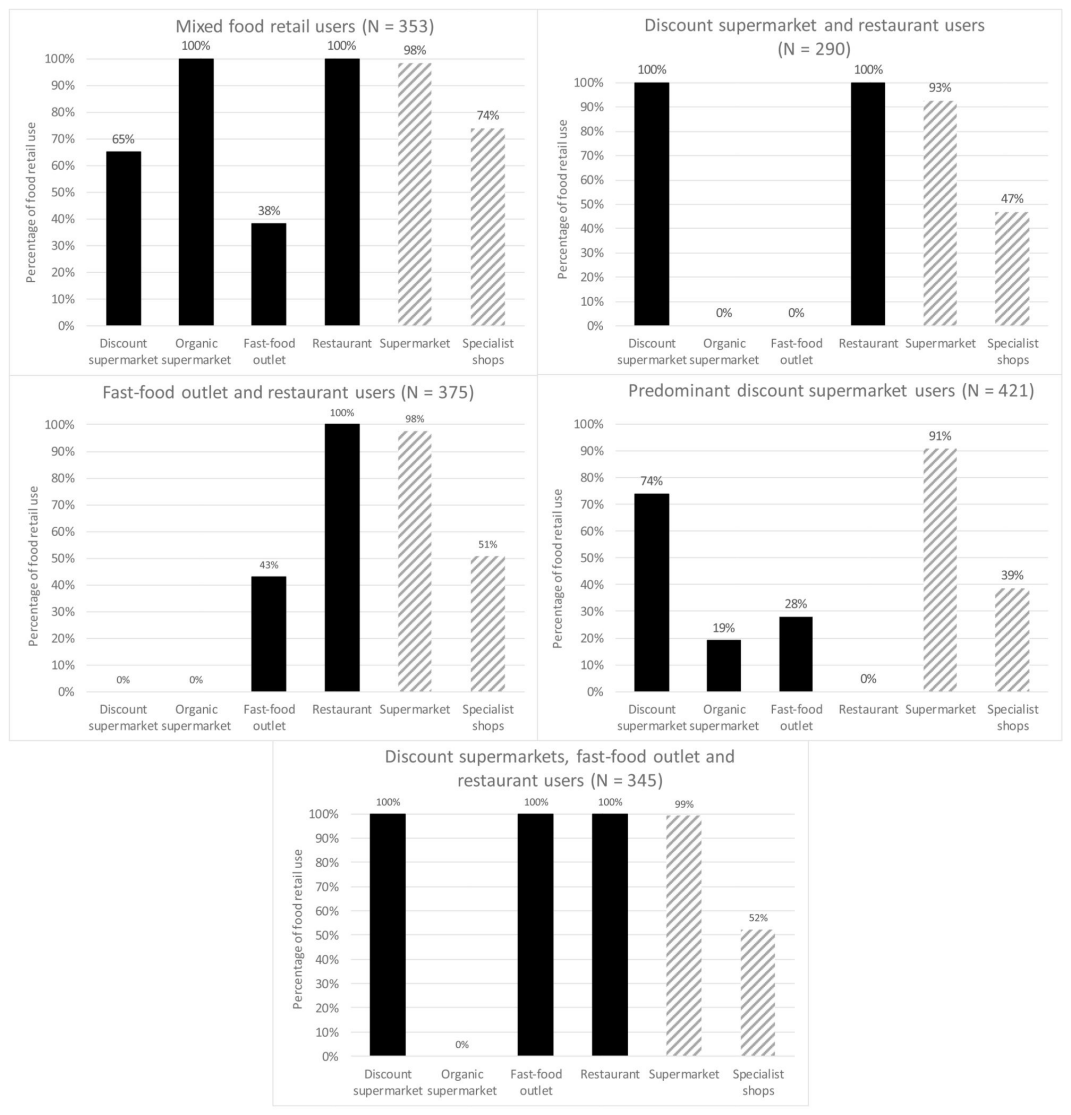
Percentage of participants (N = 1784) in the clusters who used food retailers Note: The striped bars represent the food retail variables that were not included in the two-step cluster analysis due to a low predictor importance. Nevertheless, as these food retailers are still used by the participants included in these clusters, they were also included in the figure.

Meaningful clustering was only found when the food retail variables within a 10-minute walk and further from home were combined (i.e. food retail use closer to home and further from home was combined into a single measure of food retail use) and when regular supermarkets and specialist shops were excluded due to their low predictor importance. Thus, only use of discount supermarkets, organic supermarkets, fast-food outlets and restaurants were included in clusters. After manual determination, five clusters were found with a cluster quality of 0.75 and a lowest predictor importance of 0.45. Figure 3 displays the use of food retailers within the five clusters. Despite not being used in the formation of the cluster variables, supermarkets and specialist shops were included in Figure 2 to indicate that participants within all five clusters made regular use of supermarkets (90.7%-99.1%) and specialist shops (39-74%).

As shown in Figure 3, all food retailers were represented in the *mixed food retail* cluster. The smallest cluster of n=290 participants included participants that all used both discount supermarkets and restaurants, but neither organic supermarkets nor fast-food outlets. The *fast-food outlet and restaurant users* cluster included participants who all used restaurants, some used fast-food outlets and none used discount or organic supermarkets. The largest cluster (N=421) included participants of whom most used discount supermarkets, and some used organic supermarkets and fast-food outlets; none used restaurants. The last *discount supermarket*, *fast-food outlet and restaurant users* cluster was characterized by all participants using fast-food outlets, restaurants and discount supermarkets and no participants using organic supermarkets.

### Cluster differences

As shown in Table 3, age, having a partner, the number of children in the household, SEP proxies, snacking frequency, cooking frequency, ordering dinner, grocery shopping styles and diet quality all differed statistically significantly between clusters. The *mixed food retail* cluster was characterized by participants that had the highest mean age (45.2 ±12.8), a high SEP as indicated by educational level, occupation and net household income, and had the highest DHD15-index (104.6 ±16.0). Participants in the *discount supermarket and restaurant* cluster had a relatively high DHD15-index (99.0 ±17.0), snacked least often per week (Med 7.5 IQR 7.4), and cooked most often (70.7%). Participants in the *fast-food outlet and restaurant users* cluster were the youngest (Mean 40.4 ±13.2), most often had a partner (70.4%) and had the highest SEP (e.g. 69.1% had a high educational level). Participants in the *predominant discount supermarket users* cluster had the lowest SEP, had a lower DHD15-index (Mean 93.0 ±19.3) and were less likely to have a partner (57.0%). Participants in the *discount supermarket*, *fast-food outlet and restaurant* cluster had the lowest DHD15-index (Mean 90.9 ±17.6), the highest snacking frequency per week (Med 10.1 IQR 8.9), most often ordered dinner online (71.6%) and least often cooked 6 times per week (54.8%).

Table 4 shows the mean DHD15-index score in each cluster before and after adjustments for sociodemographic and diet-related characteristics. Adjustments for sociodemographic and diet-related characteristics decreased the mean DHD15-index score in all five clusters. For example, the mean DHD15-index for the *mixed food retail use* cluster was 104.6, which reduced to 93.7 after adjustments. Nevertheless, differences in DHD15-index between clusters remained approximately the same after adjustments. Participants in the *mixed food retail use* cluster had the highest diet quality while participants in the *discount supermarket*, *fast-food outlet and restaurant cluster* had the poorest diet quality.

### Additional analysis

As we expected the cluster development to be based on a distinction between the use of food retailers within a 10-minute walk (i.e. in the residential neighbourhood environment) and further away from home, we additionally investigated how the presence and use of food retailers within the residential neighbourhood was distributed among the clusters. The results of this additional analysis displayed in Supplementary Table 4 indicate that between 8% (supermarkets) and 66% (fast-food outlets) of participants stated that certain food retailers were present in their neighbourhood, but that they chose not to use them. Despite 80% of participants stating that there are specialist shops within a 10-minute walk, only 34% of participants use these, while 42% of participants use specialist shops further away from home. Usage when being available varied between clusters, with higher percentages of non-use despite presence within a 10-minute walk in clusters with no or low use of the food retailer. Results also indicated that the vast majority of participants (i.e. 73% or more) reported to have supermarkets, specialist shops, fast-food outlets and restaurants within a 10-minute walk. Also, supermarkets were used both close to home as well as further away. Namely, 85% of participants used supermarkets within a 10-minute walk and 80% used supermarkets further away. Fast-food outlets within a 10-minute walk were often not used despite being present in the neighbourhood (between 42% and 78% of participants reported not using fast-food outlets within a 10-minute walk).

## Discussion

In this study we address an important gap in the food environment-food choice literature around the individual characteristics associated with food retail usage. We sought to understand to what extent there are distinct clusters of food retail use among Dutch adults. We identified five distinct clusters: 1) *mixed food retail users*, 2) *discount supermarket and restaurant users*, 3) *fast-food outlet and restaurant users*, 4) *predominant discount supermarket users*, and 5) *discount supermarket, fast-food outlet and restaurant users*. The distinction between the use of food retailers close to home and further away did not contribute to the final cluster solution. Furthermore, including supermarkets and specialty shops did not lead to meaningful clusters, likely because they were used by at least some proportion of participants in each cluster. As such, these food retailers were excluded from the final cluster solution. The five clusters consisted of individuals with varying sociodemographic and diet-related characteristics. For example, individuals in the cluster with the second highest diet quality (i.e. *discount supermarket and restaurant users*) generally had healthier diet-related behaviours in that they snacked the least, had the highest frequency of cooking 6-7x a week and most often did the grocery shopping for a week. The individuals in the *discount supermarket, fast-food outlet and restaurant users* cluster with the lowest diet quality, had the highest snacking frequency, a relatively low SEP and the lowest cooking frequency. Interestingly, whether or not a food retailer in the residential neighbourhood (i.e. within a 10-minute walk from home) is used seems to be related to the type of food retailer.

The current study found that use of regular supermarkets was highly prevalent and therefore did not contribute to the clustering of food retail use. Similarly, previous US-based clustering studies found that most individuals, including those with strong preferences for supermarkets, also use a variety of additional food retailers [21, 22]. Also, the study by Yenerall et al. [22] found that the mixed food retail cluster consisted of participants with more children in the household and a higher income compared to the cluster dominated by superstores and supermarkets. While our *mixed food retail* cluster included participants with a relatively high SEP, it did not necessarily differ in terms of presence of children in the household compared to for example the *discount supermarket and restaurant* cluster. Comparing clusters and the participant characteristics of the clusters with previous studies is difficult due to, among other factors, differences in the types of food retailers prevalent in different countries and considered in previous analyses. The different food retailers included in the studies are likely a result of contextual differences between the US and the Netherlands. Unlike the US, the Netherlands does not have superstores, and instead supermarkets are mostly distinguishable based on product price (e.g. discount vs regular), product range (e.g. regular vs discount) and product variety (e.g. organic/discount vs regular).

Previous research suggests that socio-demographic characteristics are associated with food retail use as well as distance traveled [11, 16, 42, 43]. For example, there is evidence to suggest that lower SEP is associated with more fast-food exposure, fast-food use, and (discount) supermarket use [11, 16, 42]. Also, older age and being male is associated with use of discount supermarkets [11]. As such, the sociodemographic and diet-related characteristics of the current study population likely led to the discovery of these specific five clusters [23].

The specific clusters found in this study may not be generalizable to other contexts due to differences in sociodemographic characteristics, types of food retailers available as well as other factors such as culture. Nonetheless, the mere presence of food retail clusters as well as the other study results have important implications for future studies aiming to investigate the role of the food environment on diet and obesity. For example, our results suggest that clustering of food retail use is independently associated with diet quality since adjustments for sociodemographic and diet-related characteristics did not influence the ranking of participants' dietary quality across clusters. The comparatively high diet quality found in the *mixed food retail* cluster, in spite of few menu offerings from popular fast-food chains meeting the recommended dietary guidelines [44], may be because the consumption of foods from fast-food outlets and restaurants are offset by healthier food purchases and consumption from organic supermarkets and specialist shops. Indeed, a recent report suggests that a popular organic supermarket chain in the Netherlands has a healthier product assortment compared to regular and discount supermarkets [45]. Thus, shopping at the organic supermarket across the street may lead to healthier food purchases. However, individuals preferring healthier foods may be more likely to use a food retailer that provides these healthier foods (e.g. organic supermarkets). In other words, the relationship between environmental exposure and dietary behaviours is likely dynamic; with causation flowing in both directions and through positive or negative feedback loops [46]. It was not our aim to determine the direction of causation between food retail cluster use and diet quality, but rather to investigate if there is an association between the two. Future longitudinal and experimental studies should explore direction(s) of causation between food retail exposure/use and diet quality, and the potential feedback loops.

Another interesting finding was that despite food retailers being present in the residential neighbourhood, this did not necessarily imply that they were used (except in the case of regular supermarkets). For example, despite 85% of participants saying they had a fast-food outlet within 10-minute walk, 66% of participants indicated that they did not use these, and 38% of participants stated that they used fast-food outlets further away from home. While the reporting of fast-food outlet use may be sensitive to social desirability bias, and fast-food outlets may be used near other settings where individuals spend a lot of time (e.g. the workplace), these results may also imply that people are selective in their use of food retailers, and again it is possible that the availability of a specific food retailer does not necessarily lead to use. It is likely that a combination of individuals' desire to consume foods and food retail characteristics lead to use of food retailers [47]. The Netherlands is a highly dense country with excellent infrastructure (especially in urban areas where most participants were recruited). Due to this limited variation in proximity to food retailers (e.g. more than 73% of participants reported to have supermarkets, specialist shops, fast-food outlets and restaurants within a 10-minute walk), it is possible that the willingness and opportunity to travel a bit further for a preferred food retailer, and other factors such as pricing [48, 49], food quality [49], convenience, and family preferences [48] play important roles in food retail usage. Even in more rural areas/countries, residential exposure to food retailers may not lead to usage due to for example trip chaining (e.g. stopping at the supermarket between work and home) [16, 50].

The present study showed that people 1) use a variety of food retailers and 2), with the exception of supermarkets, generally do not use food retailers close to home. Our work thereby challenges the common methodological choice of using data on physical proximity to food retailers as a proxy of shopping behaviour as this might not be an accurate metric of exposure (especially in the Netherlands where there is little variation in proximity to food retailers). The finding that people do not necessarily use food retailers close to home (except for supermarkets) may partly explain the inconsistent associations between local food environments, dietary intake and obesity [6, 7]. While the current findings do not imply that the residential food environment does not influence food retail usage, it does highlight the need to incorporate food shopping patterns into future research and policy [23]. To make progress in the food environment-behaviour literature, drivers of food retail use and its consequences on dietary behaviours and health must be untangled before placing too much policy focus on physical food retail exposure alone. For example, a supermarket may be available to those living around it, but they may not use that supermarket for a variety of reasons: because it is too expensive, because food shopping is done near the work environment and/or personal preferences [11, 51]. Thus, intervening on the physical location of food retailers only is unlikely to be sufficient for achieving substantial improvements in diet quality. Different interventions implemented simultaneously will likely lead to substantial diet quality improvements, such as offering lower prices for healthy relative to less healthy foods, restricting the marketing of unhealthy foods and promoting healthy foods [23].

The strengths and limitations of this study should be considered when interpreting the results. The first strength of the study is that, as far as we are aware, it is the first study to investigate clustering of food retail use outside the US. Furthermore, different types of supermarkets were differentiated, allowing for additional insights into mechanisms behind food purchases and diet quality [11]. A third strength is that robustness checks were performed on the cluster solution, which led to the same cluster solution both before and after the checks. Lastly, we included a relatively large sample size of participants recruited from different urban regions in the Netherlands, likely making our results generalizable to the population living in this area. Indeed, participant characteristics with regards to age, having a partner and number of children in the household are comparable to the study sample of the Dutch National Food Consumption Survey 2012-2016 (DNFCS) which recruited a representative sample of the Dutch population [52]. The DNFCS also found that approximately 50% of Dutch people live in an extremely or strongly urbanized region, making the current clustering results generalizable to almost half of the Dutch population. Nevertheless, as previously discussed, differences in contextual factors can hinder the generalizability of the clustering results to other areas in the Netherlands as well as other countries. A limitation includes the self-reported data which may be subject to social desirability bias, resulting in for example under reportage of dietary intake [53]. Also, while the current study findings may suggest that proximity is not an important distinguishing feature of food retail use, this cannot be inferred due to the crude nature of the proximity variable (i.e. within 10 minutes walk of home or further away). It also does not capture proximity from other locations (e.g. work [17]). Other limitations include the fact that participants could only select from a list of six food retailers, that the proportion of daily or weekly food consumption from each food retailers was not measured and that only usage by participants themselves were reported (and not that of partners or housemates).

## Conclusion

This study shows that Dutch adults use a variety of food retailers and that food retail use is clustered. Differences were found between participants in the clusters, in particular regarding their diet quality, SEP, snack intake, cooking frequency and meal delivery. Generally, use of food retailers further from home was more common than within the residential neighbourhood (except for supermarkets), indicating that the availability of a specific food retailer does not necessarily lead to use. It is important to understand the patterning of food retail usage, as it increases our understanding of the ways in which food environments influence food choices and obesity.

## Supplementary Material

Supplementary Tables

## Figures and Tables

**Table 1:** Questions, answering options and coding relating to food retail usage

Question	Food retailers	Answer	Coding
1. Are the following retailers present in your area within a 10-minute walk?	Regular supermarkets	Yes, I go there regularly	User; within 10-minute walk
	Organic supermarkets		
	Discount supermarkets	Yes, but I rarely go there	Non-user; within 10-minute walk
	Specialist shops		
	Fast-food outlets	No	Non-user; further away from home
	Restaurants		
2. Do you use facilities while they are a bit further away	Regular supermarkets	Yes	User; further away from home
	Organic supermarkets		
	Discount supermarkets		
	Specialist shops	No	Non-user; further away from home
	Fast-food outlets		
	Restaurants		

**Table 2:** Sociodemographic characteristics and diet-related behaviours of the analytical (N = 1784)

Sociodemographic characteristics		n	Mean (SD), median (IQR) or n (%)
Age in years (SD)		1784	42.5 (13.7)
Gender (% women)		1784	1137 (63.7)
Partner (% yes)		1784	1182 (66.3)
Number of children living in the household (%)	0	1784	1054 (59.1)
	1		266 (14.9)
	2 or more		464 (26.0)
Educational level (%)	Low	1778	200 (11.2)
	Medium		574 (32.3)
	High		1004 (56.5)
Occupation (%)	Skill level 1	1665	54 (3.2)
	Skill level 2		537 (32.3)
	Skill level 3		326 (19.6)
	Skill level 4		748 (44.9)
Net monthly household income (%)	€0 - €1200	1639	201 (12.3)
	€1200 - €1800		195 (11.9)
	€1800 - €2600		353 (21.5)
	€2600 - €4000		489 (29.8)
	More than €4000		401 (24.5)
**Diet-related behaviours**		**n**	**Mean (SD), median (IQR) or N (%)**
Median snacking frequency per week (IQR)		1784	8.6 (7.7)
How often do you (or your partner) cook at home?	6-7x a week	1784	1111 (62.3)
	3-5x a week		557 (31.2)
	2x a week or less		116 (6.5)
Do you sometimes purchase groceries online? (% yes)^1^		1784	494 (27.7)
How often do you order dinner online (% never)^A^		1784	648 (36.3)
Before I go grocery shopping, I (or my partner) make a grocery list (%)	Always	1784	629 (35.3)
	Very often		585 (32.8)
	Sometimes		274 (15.4)
	Rarely or never		296 (16.6)
I (or my partner) go to the grocery store approximately once a week (%)	Always	1784	287 (16.1)
	Very often		523 (29.3)
	Sometimes		292 (16.4)
	Rarely or never		682 (38.2)
I decide in the grocery store what I will purchase (%)	Always	1784	40 (2.2)
	Very often		296 (16.6)
	Sometimes		627 (35.1)
	Rarely or never		821 (46.0)
**Diet quality**		**n**	**Mean (SD)**
DHD15-index (SD)^B^		1461	96.3 (18.3)

**DHD15-index:** *Dutch Healthy Diet index 2015*

SD *Standard deviation*

IQR *Interquartile range*

A *Participants that indicated to never do online grocery shopping or use meal delivery services were coded as ‘no’, and those that indicated to do online grocery shopping or use meal delivery services 1-2x per year to almost every day were coded as ‘yes’.*

B *DHD15-index Dutch Healthy Diet 2015 index (DHD15-index) score ranging from 0 to 150*

**Table 3:** Sociodemographic and diet-related characteristics of participants in clusters (N = 1784)

		Mixed food retail users (n = 353)	Discount supermarket and restaurant users (n = 290)	Fast-food outlet and restaurant users (n = 375)	Predominant discount supermarket users (n = 421)	Discount supermarket, fast-food outlet and restaurant users (n = 345)	Chi-value (df), F value (df) or H value (df)*
**Socio-demographic characteristics**							
Age in years (SD)		45.2 (12.8)^BCE^	42.2 (13.6)^A^	40.4 (13.2)^AC^	43.9 (13.8)^CE^	41.6 (13.5)^AD^	8.5 (4)^≠^
Sex N (%) women		236 (66.9)	191 (65.9)	232 (61.9)	263 (62.5)	215 (62.3)	3.2 (4)
Partner N (%) yes		239 (67.7)^D^	199 (68.6)^D^	264 (70.4)^D^	240 (57.0)^ABCE^	240 (69.6)^D^	21.7 (4)^≠^
Number of children in N (%)	0 children	214 (60.6)	177 (61.0)	231 (61.6)	244 (58.0)	188 (54.5)	15.7 (8)^₸^
	1 child	62 (17.6)	34 (11.7)	61 (16.3)	61 (14.5)	48 (13.9)	
	2+ children	77 (21.8)^BCDE^	79 (27.2)^AE^	83 (22.1)^AE^	116 (27.6)^AE^	109 (31.6)^ABCD^	
Educational level in N (%)^F^	Low	20 (5.7)	20 (6.9)	12 (3.2)	91 (21.7)	57 (16.5)	134.3 (8)^≠^
	Medium	98 (27.9)	93 (32.2)	104 (27.7)	157 (37.5)	122 (35.4)	
	High	233 (66.4)^DE^	175 (60.8)^CDE^	259 (69.1)^BDE^	171 (40.8)^ABCE^	166 (48.1)^ABCD^	
Occupation in N (%)^F^	Skill level 1	4(1.2)	6(2.2)	6(1.7)	22 (5.8)	16 (5.0)	85.6 (12)^≠^
	Skill level 2	82 (24.2)	82 (29.4)	84 (24.1)	160 (41.9)	129 (40.7)	
	Skill level 3	69 (20.4)	63 (22.6)	64 (18.4)	69 (18.1)	61 (19.2)	
	Skill level 4	184 (54.3)^BDE^	128 (45.9)^ACDE^	194 (55.7)^BDE^	131 (34.3)^ABC^	111 (35.0)^ABC^	
Net monthly household income in N (%)^F^	€0 - €1200	23 (6.9)	23 (9.0)	24 (6.8)	92 (24.5)	39 (12.2)	143.3 (16) ^≠^
	€1200 - €1800	40 (11.9)	20 (7.8)	31 (8.8)	61 (16.2)	43 (13.5)	
	€1800 - €2600	65 (19.4)	62 (24.2)	63 (17.8)	87 (23.1)	76 (23.8)	
	€2600 - €4000	100 (29.9)	89 (34.8)	109 (30.9)	94 (25.0)	97 (30.4)	
	More than €4000	107 (31.9) ^BDE^	62 (24.2)^ACD^	126 (35.7) ^BDE^	42 (11.2)^BCDE^	64 (20.1)^ACD^	
**Diet-related behaviours**							
Median snacking frequency per week (IQR)		8.1 (7.6)^E^	7.5 (7.4)^CE^	8.5 (7.5)^BDE^	7.8 (7.8)^CE^	10.1 (8.9)^ABCD^	37.8 (4)^≠^
How often do you (or your partner) cook at home?	6-7x a week	231 (65.4)^BE^	205 (70.7)^ACDE^	226 (60.3)^BE^	260 (61.8)	189 (54.8)^ABCD^	22.3 (8)^₸^
	3-5x a week	102 (28.9)^BE^	70 (24.1)^ACDE^	124 (33.1)^BE^	127 (30.2)	134 (38.8)^ABCD^	
	<2x a week	20 (5.7)	15 (5.2)	25 (6.7)	34 (8.1)	22 (6.4)	
Do you sometimes purchase groceries online? (% yes)^1^		101 (28.6)	68 (23.4)	121 (32.3)	104 (24.7)	100 (29.0)	8.8 (4)
How often do you order dinneronline (% never)^A^		213 (60.3)	176 (60.7)	277 (73.9)^ABDE^	223 (53.0)^ABCE^	247 (71.6)^ABCD^	49.8 (4)^≠^
Before I go grocery shopping, I (or my partner) make a grocery list (%)	Always	112 (31.7)	99 (34.1)	155 (41.3)	159 (37.8)	104 (30.1)	25.0 (16)
	Very often	122 (34.6)	113 (39.0)	112 (29.9)	126 (29.9)	112 (32.5)	
	Sometimes	61 (17.3)	40 (13.8)	51 (13.6)	60 (14.3)	62 (18.0)	
	Rarely/never	58 (16.4)	38 (13.1)	57 (15.2)	76 (18.0)	67 (19.5)	
I (or my partner) go to the grocery store approximately once a week (%)	Always	36 (10.2)^BCDE^	59 (20.3)^ACDE^	68 (18.1)^AB^	66 (15.7)^AB^	58 (16.8)^AB^	52.2 (16)^≠^
	Very often	98 (27.8)	81 (27.9)	114 (30.4)	112 (26.6)	118 (34.2)	
	Sometimes	67 (19.0)^C^	50 (17.2)^C^	32 (8.5)^ABDE^	80 (19.0)^C^	63 (18.3)^C^	
					
	Rarely/never	152 (45.1)^BE^	100 (34.5)^ACDE^	161 (43.0)^BE^	163 (39.7)^BE^	106 (30.7) ^ABCD^	
I decide in the grocery store what I will purchase (%)	Always	9(2.5)^E^	4(1.4)^E^	5(1.3)^E^	9(2.1)^E^	13 (3.8)^ABCD^	34.5 (16)^≠^
	Very often	57 (16.1)	44 (15.2)	68 (18.1)	75 (17.8)	52 (15.1)	
	Sometimes	134 (38.0)^DE^	103 (35.5)^DE^	122 (32.5)^DE^	131 (31.1)^ABCE^	137 (39.7)^ABCD^	
	Rarely/never	153 (43.4)^DE^	139 (47.9)^DE^	180 (48.0)^D^	206 (49.0)^ABE^	143 (41.5)^ABCD^	
DHD15-index (SD)^F^		104.6 (16.0)^BCDE^	99.0 (17.0)^ACDE^	95.3 (18.1)^ABE^	93.0 (19.3)^AB^	90.9 (17.6)^ABC^	26.1 (4)^≠^

A *statistically significantly different from mixed food retail cluster*

B *statistically significantly different from discount supermarket and restaurant cluster*

C *statistically significantly different from fast-food outlet and restaurant cluster*

D *statistically significantly different from predominant discount supermarket cluster*

E *statistically significantly different from discount supermarket, fast-food outlet and restaurant cluster*

F *n_educational level_ = 1778, n_occupation_ = 1665, n_net household income_ = 1639, n_DHD15-index_ =1461*

₸ *p-value smaller than 0.05*

≠ *p-value smaller than 0.01*

* *Based on Chi-Squared tests (in the case of categorical variables), analyses of variance (ANOVA in case of normally distributed continuous variables)) or the Kruskal-wallis H test (in case of non-normally distributed continuous variables)*

SD *Standard deviation*

IQR *Interquartile range*

**Table 4:** General Linear Model results of the mean diet quality as measured by the DHD15-index of participants in the clusters adjusted for covariates Model 0 is unadjusted, Model 1 is adjusted for sociodemographic characteristics (i.e. age, sex, partner, number of children, net household income, educational level and occupation), and model 2 is adjusted for both sociodemographic and diet-related characteristics

	Model 0^F^	Model 1^G^	Model 2^G^
Clusters		Mean (95%CI) DHD15-index	
Mixed food retail users (n = 279)	104.6 (102.5; 106.7)^BCDE^	97.6 (95.0; 100.2)^BCDE^	93.7 (90.5; 96.9)^BCDE^
Discount supermarket and restaurant users (n = 243)	99.0 (96.7; 101.2)^ACDE^	93.2 (90.4; 95.9)^ACDE^	88.9 (85.6; 92.3)^ACDE^
Fast-food outlet and restaurant users (n = 317)	95.3 (93.3; 97.2)^ABE^	89.2 (86.7; 91.7)^AB^	85.8 (82.7; 89.0)^AB^
Predominant discount supermarket users (n = 344)	93.0 (91.1; 94.9)^AB^	89.0 (86.7; 91.3)^AB^	84.7 (81.6; 87.7)^AB^
Discount supermarket, fast-food outlet and restaurant users (n = 278)	90.9 (88.9; 93.0)^ABC^	87.2 (84.8; 89.7)^AB^	84.0 (81.0; 87.1)^AB^

A *statistically significantly different from mixed food retail cluster*

B *statistically significantly different from discount supermarket and restaurant cluster*

C *statistically significantly different from fast-food and restaurant cluster*

D *statistically significantly different from predominant supermarkets cluster*

E *statistically significantly different from discount supermarket, fast-food outlet and restaurant cluster*

F *n=1461*

G *n=1260 due to missing data in the SEP proxies. The cluster sizes are mixed food retail cluster n = 254, discount supermarket and restaurant cluster n = 204, fast-food outlet and restaurant cluster n = 282, predominant supermarkets cluster n = 278, discount supermarket, fast-food outlet and restaurant cluster n = 242.*

## Data Availability

The datasets used during the current study are available from JDM on reasonable request.

## List of abbreviations

BIC: Bayesian Information Criterion
DHD15-index: Dutch Healthy Diet index 2015
DNFCS: Dutch National Food Consumption Survey 2012-2016
SEP: Socioeconomic Position
US: United States
