# Supplementary material for: Who uses what food retailers? A cluster analysis of food retail usage in the Netherlands
Source: Health Place. Author manuscript; Available in PMC 2023 Jun 6. (PMC7614624; doi:10.1016/j.healthplace.2023.103009)
Supplement: Supplementary Tables [file EMS176252-supplement-Supplementary_Tables.docx]

Supplementary Table 1. Questions used to assess snacking frequency in Dutch with free English translations between brackets

| Hoe vaak eet u…. (how often do you consume) | Nooit (never) | 1-2x per jaar (1-2x per year) | 5-6x per jaar (5-6x per year) | 1x per maand (once per month) | 2x per maand (twice per month) | 1x per week (once per week) | 2x per week (twice per week) | 3x per week (three times per week) | 4x per week (four times per week) | 5x per week (five times per week) | 6x per week (six times per week) | elke dag (every day) |
| --- | --- | --- | --- | --- | --- | --- | --- | --- | --- | --- | --- | --- |
| …snoep? (candy) |  |  |  |  |  |  |  |  |  |  |  |  |
| …chocolade? (chocolate) |  |  |  |  |  |  |  |  |  |  |  |  |
| …koekjes? (cookies) |  |  |  |  |  |  |  |  |  |  |  |  |
| …cake of taart? (cake) |  |  |  |  |  |  |  |  |  |  |  |  |
| …ijs? (ice cream) |  |  |  |  |  |  |  |  |  |  |  |  |
| … chips of zoutjes zoals kaasvlinders, paprikachips of zoute sticks? (crisps or other savoury snacks) |  |  |  |  |  |  |  |  |  |  |  |  |
| … nootjes zoals pinda’s, borrelnootjes of amandelen? (nuts such as peanuts) |  |  |  |  |  |  |  |  |  |  |  |  |
| …pizza? (pizza) |  |  |  |  |  |  |  |  |  |  |  |  |
| … gefrituurde snacks zoals patat, kroket of bamihap? (fried snacks such as French fries) |  |  |  |  |  |  |  |  |  |  |  |  |
| … saucijzenbroodjes, hotdogs of worstenbroodjes? (sausage bread and hot dogs) |  |  |  |  |  |  |  |  |  |  |  |  |

Supplementary Table 2. Questions assessing how often participants cook and whether they order evening meals online in Dutch with free English translations between brackets

| En hoe vaak…. (and how often) | Nooit (never) | 1-2x per jaar (1-2x per year) | 5-6x per jaar (5-6x per year) | 1x per maand (once per month) | 2x per maand (twice per month) | 1x per week (once per week) | 2x per week (twice per week) | 3x per week (three times per week) | 4x per week (four times per week) | 5x per week (five times per week) | 6x per week (six times per week) | elke dag (every day) |
| --- | --- | --- | --- | --- | --- | --- | --- | --- | --- | --- | --- | --- |
| …kookt u of uw partner thuis? (do you or your partner cook at home) |  |  |  |  |  |  |  |  |  |  |  |  |
| …bestelt u een avondmaaltijd online? (do you order dinner online) |  |  |  |  |  |  |  |  |  |  |  |  |

Supplementary Table 3. Questions used to assess grocery shopping style in Dutch with free English translations between brackets

|  | **Altijd (always)** | **Meestal (most often)** | **Soms (sometimes)** | **Zelden (rarely)** | **Nooit (never)** |
| --- | --- | --- | --- | --- | --- |
| **Voordat ik boodschappen ga doen, maak ik of mijn partner een boodschappenlijstje (before I do the grocery shopping, my partner or I make a shopping list)** |  |  |  |  |  |
| **Ik of mijn partner doe ongeveer één keer in de week de boodschappen voor de hele week (my partner or I do the weekly grocery shopping once a week)** |  |  |  |  |  |
| **Ik beslis pas in de winkel wat ik ga kopen (only in the store do I decide what I will purchase)** |  |  |  |  |  |

Supplementary Table 4. Percentage of presence and use of food retailers within a 10-minute walk from the home and use of food retailers further away from home by clusters

|  | |  | Mixed food retail users  (N = 353) | Discount supermarket and restaurant users  (N = 290) | Fast-food outlet and restaurant users  (N = 375) | Predominant discount supermarket users  (N = 421) | Supermarket, outlet and restaurant users  (N = 345) | Total |
| --- | --- | --- | --- | --- | --- | --- | --- | --- |
| Discount supermarket | Presence and use in 10-minute walk | Yes, I go there frequently | 35.8% | 54.7% | 0.0% | 48.8% | 54.7% | 37.9% |
|  |  | Yes, but I do not go | 20.1% | 7.4% | 52.7% | 19.5% | 9.0% | 22.8% |
|  |  | No | 44.1% | 37.9% | 47.3% | 31.7% | 36.3% | 39.4% |
|  | Use further away | Yes | 56.9% | 80.0% | 0.0% | 61.0% | 90.1% | 56.1% |
|  |  | No | 43.1% | 20.0% | 100.0% | 39.0% | 9.9% | 43.9% |
| Supermarket | Presence and use in 10-minute walk | Yes, I go there frequently | 88.2% | 83.5% | 88.0% | 78.2% | 85.6% | 84.5% |
|  |  | Yes, but I do not go | 5.4% | 8.6% | 8.5% | 12.2% | 6.1% | 8.4% |
|  |  | No | 6.5% | 7.8% | 3.5% | 9.6% | 8.3% | 7.1% |
|  | Use further away | Yes | 83.0% | 78.6% | 78.7% | 73.4% | 89.0% | 80.3% |
|  |  | No | 17.0% | 21.4% | 21.3% | 26.6% | 11.0% | 19.7% |
| Organic supermarket | Presence and use in 10-minute walk | Yes, I go there frequently | 44.8% | 0.0% | 0.0% | 8.1% | 0.0% | 10.5% |
|  |  | Yes, but I do not go | 7.9% | 35.4% | 36.9% | 26.5% | 24.1% | 26.2% |
|  |  | No | 47.3% | 64.6% | 63.1% | 65.4% | 75.9% | 63.3% |
|  | Use further away | Yes | 85.3% | 0.0% | 0.0% | 15.0% | 0.0% | 20.4% |
|  |  | No | 14.7% | 100.0% | 100.0% | 85.0% | 100.0% | 79.6% |
| Local food shop | Presence and use in 10-minute walk | Yes, I go there frequently | 48.0% | 33.7% | 36.6% | 24.1% | 29.1% | 33.9% |
|  |  | Yes, but I do not go | 35.1% | 49.4% | 45.7% | 51.2% | 48.2% | 46.1% |
|  |  | No | 16.8% | 16.9% | 17.7% | 24.7% | 22.7% | 20.0% |
|  | Use further away | Yes | 58.6% | 34.5% | 40.0% | 31.6% | 44.3% | 41.6% |
|  |  | No | 41.4% | 65.5% | 60.0% | 68.4% | 55.7% | 58.4% |
| Fast-food outlet | Presence and use in 10-minute walk | Yes, I go there frequently | 20.1% | 0.0% | 18.3% | 9.9% | 43.9% | 18.5% |
|  |  | Yes, but I do not go | 67.0% | 78.2% | 68.8% | 73.3% | 42.1% | 66.0% |
|  |  | No | 12.9% | 21.8% | 12.9% | 16.9% | 14.0% | 15.5% |
|  | Use further away | Yes | 30.9% | 0.0% | 38.1% | 24.2% | 91.9% | 37.6% |
|  |  | No | 69.1% | 100.0% | 61.9% | 75.8% | 8.1% | 62.4% |
| Restaurant | Presence and use in 10-minute walk | Yes, I go there frequently | 39.1% | 22.2% | 35.6% | 0.0% | 26.6% | 24.0% |
|  |  | Yes, but I do not go | 40.5% | 44.0% | 41.3% | 70.1% | 39.2% | 48.0% |
|  |  | No | 20.4% | 33.7% | 23.0% | 29.9% | 34.2% | 28.1% |
|  | Use further away | Yes | 96.3% | 95.2% | 96.5% | 0.0% | 96.8% | 73.5% |
|  |  | No | 3.7% | 4.8% | 3.5% | 100.0% | 3.2% | 26.5% |
